# Predictive coding of the statistical parameters of uncertain rewards by orbitofrontal neurons

**DOI:** 10.1016/j.bbr.2018.04.041

**Published:** 2018-12-14

**Authors:** Martin O’Neill, Wolfram Schultz

**Affiliations:** a Department of Experimental Psychology, University of Oxford, Tinsley Building, Mansfield Road, Oxford, OX1 3TA, UK; b Department of Physiology, Development and Neuroscience, University of Cambridge, Downing Street, Cambridge, CB2 3EG, UK

**Keywords:** Orbitofrontal cortex, Expected value, Reward probability, Risk, Reward, Value

## Abstract

Uncertain reward outcomes are characterised by statistical parameters that capture the numerical values of the underlying probability distributions of reward values, including the expected value, risk (variance) and probability. Here we show coding of an integrated expected value signal by single orbitofrontal neurons in response to visual cues predicting uncertain rewards. Separate subpopulations of orbitofrontal neurons predominantly code the prediction of one statistical parameter with few neurons showing combined coding. These signals are likely combined with subjective value signals to inform learning and decision making under conditions of uncertainty.

## Highlights

• Statistical parameters of uncertain outcomes (expected value, risk, probability) are coded by single neurons in the orbitofrontal cortex.
• Orbitofrontal neurons code an integrated expected value signal.
• These parameters are predominantly coded by separate subpopulations of orbitofrontal neurons.

## 1 Introduction

Decision making under conditions of uncertainty requires processing of multiple variables relating to possible outcomes. Models of decision making suggest that the first two central moments of reward probability distributions, namely expected value and risk, are key parameters for decision-making processing mechanisms. It is therefore a fundamental requirement of brain systems to accurately process these variables in order to guide efficient choices.

Blaise Pascal’s development of probability theory in the 17th century recognised that by calculating the likelihood of different outcomes from a gamble, value (*v*) and probability (*p*), an informed decision maker could choose the option that results in the maximum outcome [1]. This quantity, (*v* x *p*), known as expected value, equals the mean, average outcome from a range of possible outcomes (the first central moment).

Risk, the second central moment of reward probability distributions, captures the dispersion of the possible outcomes. This dispersion of probabilistic outcomes is typically measured as the variance or standard deviation (square root of variance). Note that risk defined in these terms is distinct from reward probability, which has a non-monotonic relationship with risk: for binary outcomes, risk is maximal when the probability of each outcome is equal to 0.5 since the certainty that an outcome will occur increases from *p* = 0.5 to *p* = 1 and the certainty that an outcome will not occur increases from *p* = 0.5 to *p* = 0 [2].

Risky information predicting reward outcomes is coded by single neurons in the orbitofrontal cortex [[3], [4], [5], [6], [7], [8]], cingulate cortex [8,9], supplementary eye field [10], anterodorsal septal region [11], striatum [12] and midbrain dopamine neurons [13].

Probabilistic information predicting reward outcomes is coded by single neurons in frontal and parietal cortical areas including the lateral intraparietal and parietal reach regions [14,15], the orbitofrontal, dorsolateral prefrontal and anterior cingulate cortices [[16], [17], [18], [19]]. Reward probability predictions are also coded by single neurons in subcortical regions including the globus pallidus and substantia nigra [20,21], lateral habenula [22], amygdala [23], dorsal striatum [24] and midbrain dopamine neurons [13].

Thus predictions of reward probability and risk are coded in the brain at the single neuron level and in a distributed fashion between interconnected cortical and subcortical areas. Single neurons in orbitofrontal cortex have been shown to code both reward probability and risk predictions in separate studies. Also, neurons in the orbitofrontal cortex have been shown to code predictions of reward values that reflect the subjective value of the reward predictions [25] and integrate reward magnitude and reward probability [7]. However it is not yet known if the activity of individual neurons in the orbitofrontal cortex predicts expected reward value through integration of probability, magnitude and risk. Therefore, the purpose of this study was to test for integrated expected value coding by single neurons in the orbitofrontal cortex. We identified subpopulations of orbitofrontal neurons that predominantly code the prediction of one statistical parameter with few neurons showing combined predictions of expected reward value, reward probability and risk. The ability of orbitofrontal neurons to code expected value, which requires integration of reward value, reward probability and reward risk, suggests that the orbitofrontal cortex is capable of local parsing of statistical information relevant for predicting uncertain reward outcomes.

## 2 Materials and methods

### 2.1 Subjects

Two adult male rhesus monkeys (Macaca mulatta), weighing 10–14 kg, were implanted, under general anesthesia, with a head holder and a stainless steel chamber on the skull to enable daily electrophysiological recordings from single neurons. All surgical and experimental procedures were performed under a Home Office License according to the United Kingdom Animals (Scientific Procedures) Act 1986.

### 2.2 Behavioural task

**Fig. 1:**
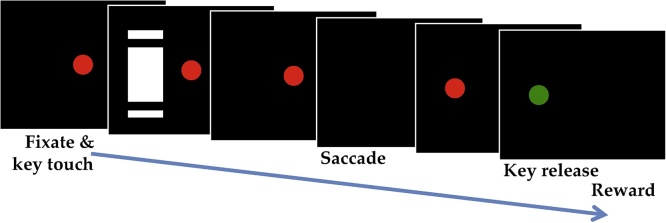
**Behavioural task**. Visual cues were presented on a monitor while monkeys fixated on a spot in the center of the screen and contacted a touch-sensitive key in front of the monitor. Only one cue was displayed per trial to the left or right of the fixation spot. When the fixation spot extinguished the monkey was required to make a saccade to the to the side where the cue was displayed. A red dot appeared in this location for 1 s before turning green, indicating that the trial was complete and the key should be released to receive a juice reward (For interpretation of the references to colour in this figure legend, the reader is referred to the web version of this article).

During training and testing, the monkeys were on a restricted water schedule 6 days of the week and 24 h water ad libitum. The monkeys were trained to sit in a restraining chair in front of a computer monitor with the head fixed and perform a memory-guided saccade task (Fig. 1). An aperture in the front of the chair provided access to a touch-sensitive key. To commence a trial, the monkey fixated on a red spot in the center of the monitor and contacted the key. After 1.5 s, a visual cue appeared in pseudorandom alternation to either the left or right of the fixation spot for 0.5 s, respectively (Table 1). The animal maintained fixation for a further 2 s before the center spot extinguished, which was the signal for the monkey to saccade to the left or right cue location. A successful saccade led to appearance of a red fixation spot at the peripheral location. After fixation for 1 s, the spot turned green and the animal released the key. Juice reward was delivered 1 s later. The next trial started with appearance of the central fixation spot at 3.5 s after the reward. Thus, intertrial interval was 3.5 s, and total cycle time (trial duration + intertrial interval) was 10.5 s. Typically, a session lasted for 600 trials in total.

**Table 1:** The trial types, visual cues and actual measures used in the experimental design. The possible juice volumes to be delivered at the end of the trial were represented by the height of horizontal black bars on a white background.

Cues:						
Expected value (ml):	0.3			0.18	0.3	0.42
Reward probability:	0.5			1		
Risk (SD):	0.03	0.06	0.12	0		

### 2.3 Stimuli and independent variables

We used black bars on framed, rectangular white backgrounds as cues. The vertical position of the bar indicated juice volume. One bar within the rectangle indicated a certain juice volume that would be delivered (p = 1). Two bars indicated that one of two possible juice volumes would be delivered with equal probability (p = 0.5 each), thus explicitly indicating the risk of the outcomes (Table 1). We used three different levels of risk while keeping the mathematical expected values (EV) of the three binary distributions of juice volumes constant, and the probability of a large or small reward on any given risk trial also a constant of 0.5 (Table 1). This differentiates reward probability on any given trial from risk as they vary independently. Risk is defined as the standard deviation (SD) of a probability distribution with:

(1) $$\text{EV}=\sum_{i=1}^{n}\left({\text{p}}_{i}*{\text{x}}_{i}\right)$$

(2) $$\text{SD}=\sqrt{\sum_{i=1}^{n}{\text{p}}_{i}*{\left({\text{x}}_{i}-\text{EV}\right)}^{2}}$$

*n* = number of possible juice volumes.

### 2.4 Neuronal recording and data analysis

We isolated and recorded the activity of single neurons in the orbitofrontal cortex while monkeys performed the task, according to procedures previously described [5]. In the first step of analysis, we defined the presence of cue-related neuronal responses by the Wilcoxon test, which compared neuronal activity during a period of 0.1–0.6 s following cue onset against a control period of 1.0 s before the fixation spot. In the second step we carried out a multiple linear regression analysis on the cue responses identified by the Wilcoxon test:

(3) Y = *β*_0_ + *β*_1_EV +  *β*_2_Probability + *β*_3_Risk + *e*

Y is neuronal firing rate, *β*_1_, *β*_2_, & *β*_3_ are corresponding regression coefficients, *β*_0_ is intercept, and *e* is error.

As the EV was the same for all three risk cues, and equal to the EV of the medium value cue (Table 1), we carried out a Tukey-Kramer post-hoc ANOVA to test whether the neuronal responses were statistically similar or different between the risk cues compared to the medium value cue.

To quantify the extent to which the regressors accounted for the variance of the neuronal data, we used the coefficient of partial determination (CPD). We also carried out a chi-square test on the cue responses to pairs of variables to test for the likelihood of combined coding of pairs of variables.

## 3 Results

We recorded the extracellular activity of 170 single neurons in the orbitofrontal cortex during task performance. Of these, 126 neurons (74%) responded significantly to the cues (*p* < 0.05, Wilcoxon test).

**Fig. 2:**
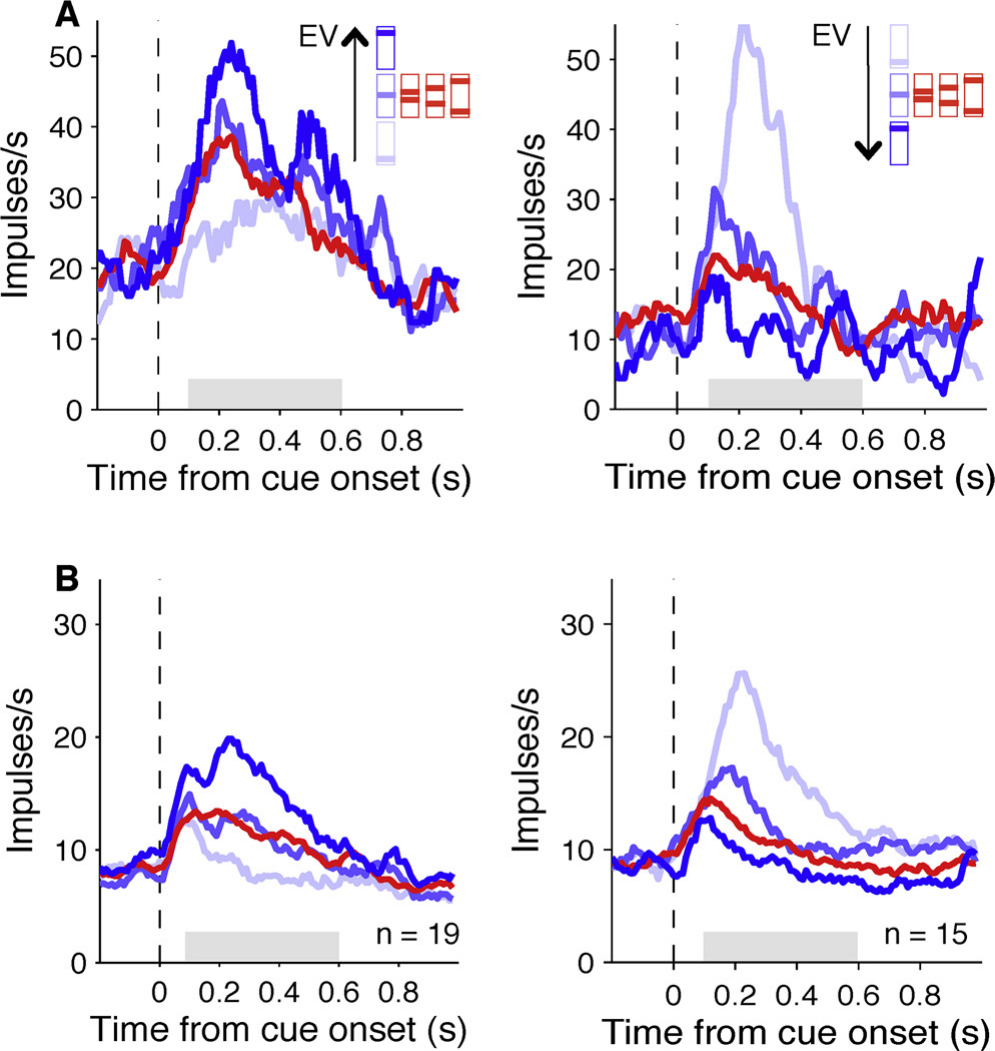
**Orbitofrontal neurons code expected value**. (A) Smoothed histograms showing examples of individual neurons coding expected value following cue onset with positive slope (left) or negative slope (right). The shaded area shows the time window used for analysis. (B) Smoothed histograms showing population responses from all neurons with statistically significant correlation coefficients for expected value during the shaded period. Risk cues with equal expected value are shown in red, and value cues with no risk are shown in blue. Colour coding of the cues in the figure legend are for presentation purposes only (For interpretation of the references to colour in this figure legend, the reader is referred to the web version of this article).

**Fig. 3:**
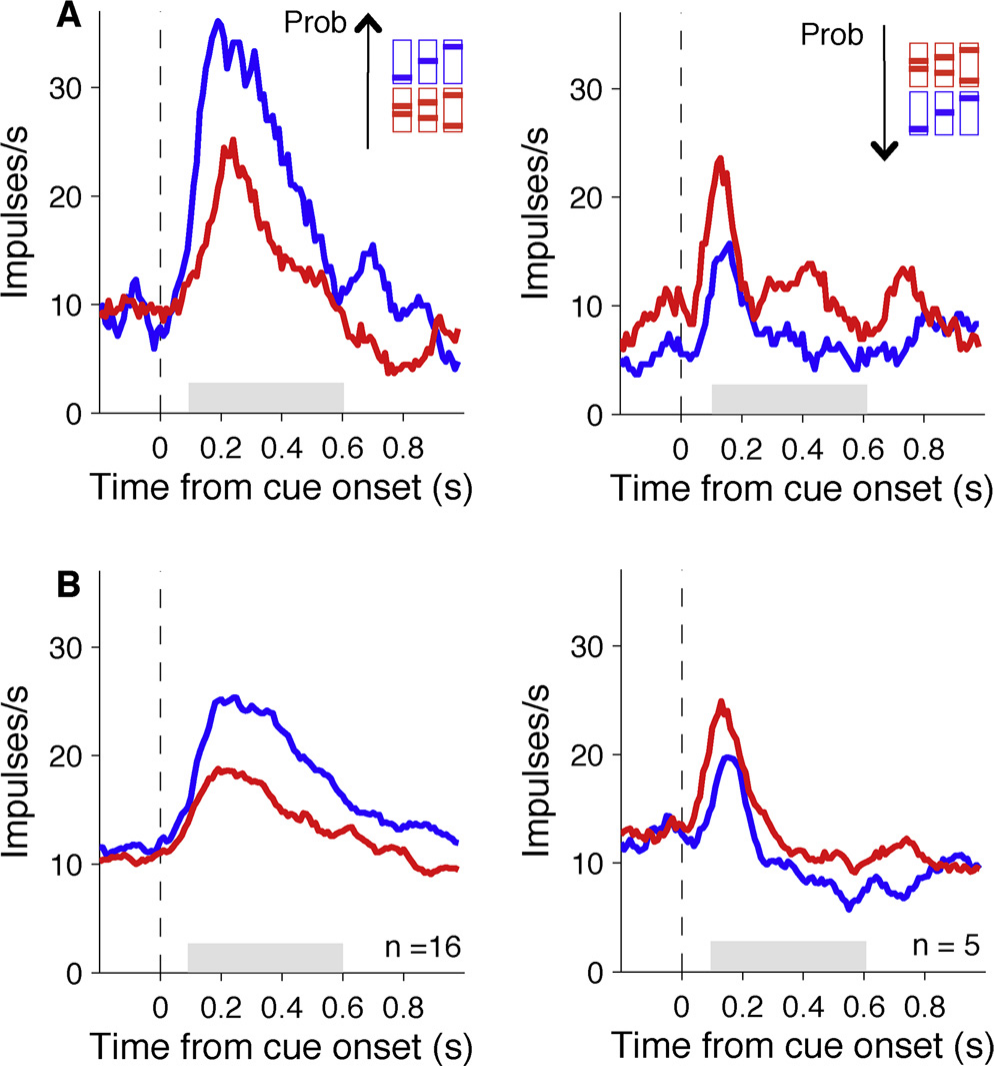
**Orbitofrontal neurons code reward probability**. (A) Smoothed histograms showing examples of individual neurons coding reward probability following cue onset with positive slope (left) or negative slope (right). The shaded area shows the time window used for analysis. (B) Smoothed histograms showing population responses from all neurons with statistically significant correlation coefficients for reward probability during the shaded period. Colour scheme the same as Fig. 2.

**Fig. 4:**
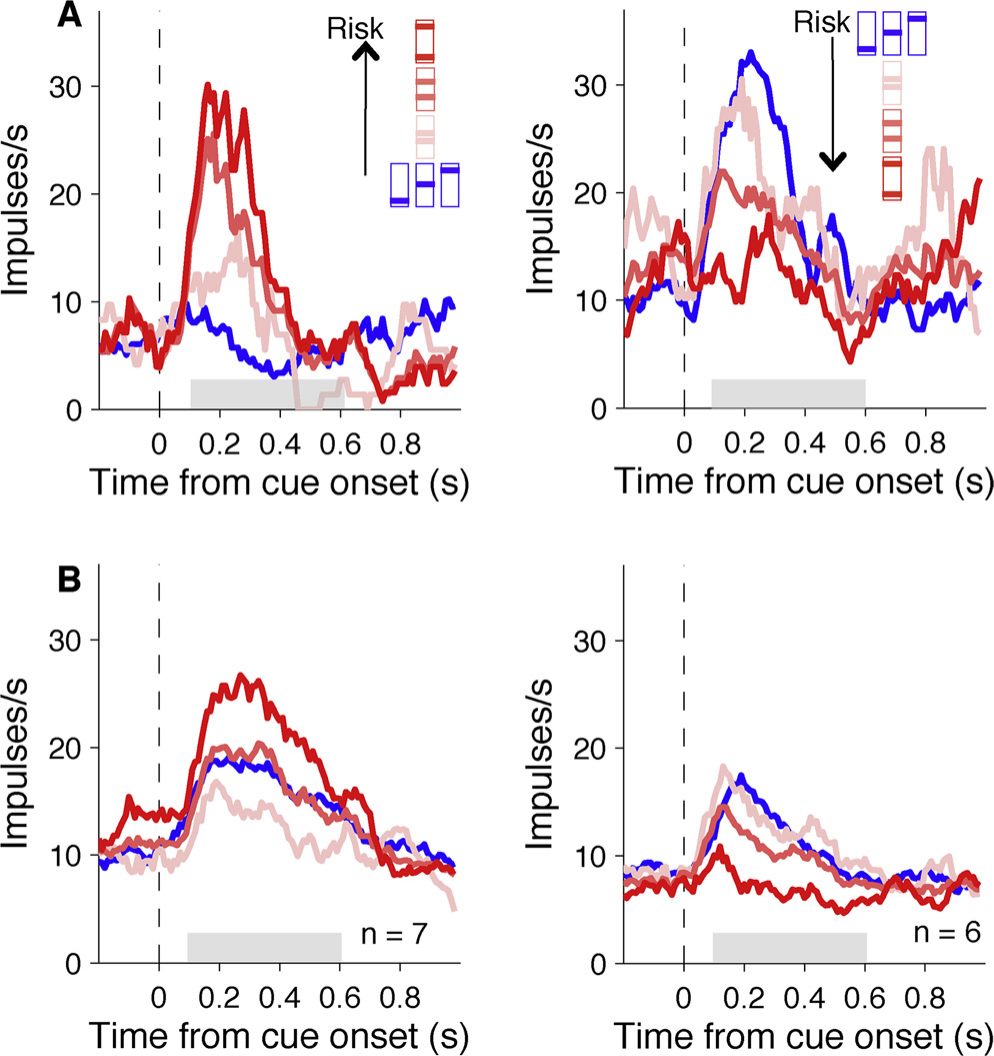
**Orbitofrontal neurons code reward risk**. (A) Smoothed histograms showing examples of individual neurons coding risk following cue onset with positive slope (left) or negative slope (right). The shaded area shows the time window used for analysis. (B) Smoothed histograms showing population responses from all neurons with statistically significant correlation coefficients for risk during the shaded period. Colour scheme the same as Fig. 2.

The multiple regression analysis (Eq. (3)) revealed that the cue responses of 42 of 126 neurons (33%) coded the EV, 21/126 coded the reward probability (17%) and 13/126 coded the risk (10%). Of the 42 neurons coding EV, 19/42 had significant positive and 23/42 had significant negative correlation coefficients (Fig. 2; all *p* <0.05). Of these neurons, the cue responses of all 19/19 neurons with positive slope and 15/23 neurons with negative slope was not significantly different between all three risk cues and the medium value cue, which were all equal in EV (left and right panels in Fig. 2, respectively; Tukey-Kramer post-hoc ANOVA test, all *p* > 0.05). Of the 21 neurons coding reward probability, 16/21 had significant positive and 5/21 had significant negative correlation coefficients (left and right panels in Fig. 3, respectively, all *p* < 0.05). Of the 13 neurons coding risk, 7/13 had significant positive and 6/13 had significant negative correlation coefficients (left and right panels in Fig. 4, respectively, all *p* < 0.05).

**Fig. 5:**
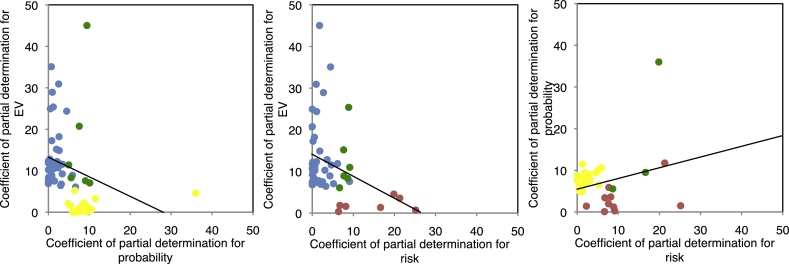
**Coefficients of partial determination (CPD) for all neurons with statistically significant correlation coefficients**. Scatterplots showing the CPD for all neurons with statistically significant correlation coefficients. Separate subpopulations of neurons largely code expected value (EV), reward probability and risk separately. Blue = neurons with significant correlation coefficients for expected value only. Yellow = neurons with significant correlation coefficients for reward probability only. Red = neurons with significant correlation coefficients for risk only. Green = neurons with significant correlation coefficients for both parameters on the x- and y-axes (For interpretation of the references to colour in this figure legend, the reader is referred to the web version of this article).

Of the 126 neurons with cue responses, 32 coded only EV, 13 coded only probability, 5 coded only risk, 5 coded both EV and probability, 5 coded both EV and risk, 2 coded both probability and risk and 1 coded all three variables. Chi-square tests on all pairs of variables were not statistically significant (all *p* > 0.05). In addition, the amount of variance explained was not positively correlated among any pair of regressors (Fig. 5; *left*, EV and probability, Pearson’s *r* = −0.26, *p* = 0.05; *middle*, EV and risk, Pearson’s *r* = −0.35, *p* = 0.01; *right*, probability and risk, Pearson’s *r* = 0.26, *p* = 0.16). Taken together, these results suggest that EV, probability and risk are coded by distinct subpopulations of neurons in this neuronal population.

## 4 Discussion

This study investigated the representation of statistical parameters relating to reward predictive coding in the orbitofrontal cortex. We show that single neurons code the expected value, the risk (variance) and the reward probability at the time of visual cues, predominantly by separate subpopulations of neurons. The experimental design intentionally facilitated orthogonalisation of the independent variables, thereby enhancing the likelihood of observing separate coding, and alternative approaches could be used in future to facilitate detection of interactions between these variables. Nonetheless, these findings advance on our previous work, showing a categorical distinction between value and risk coding [26], by showing that OFC neurons code an integrated expected value signal.

In the 17th century Blaise Pascal first proposed that decision makers perform a mental calculation to derive the expected value of uncertain, risky outcomes in order to make informed decisions. Here we show that Pascal’s proposition is indeed reflected in the brain at the level of single neurons in the orbitofrontal cortex. The observation of expected value coding to risk cues in this study is of particular interest because the expected reward value, measured in millilitres of juice (0.3 ml), is never experienced in those risk trials. Nonetheless, the neuronal activity at the time of the cue reflects this never-experienced value. This implies a predictive neuronal signal that represents a theoretical reward value associated with the visual stimuli. Expected value, risk and reward probability information must be constructed a priori through learning to represent the distribution of possible outcomes. These signals together are necessary for maintaining and updating accurate expectations of uncertain reward outcomes.

### 4.1 How are these calculations performed?

It is not yet clear how these signals are generated through the learning process. Cleary stimulus-reward associations occur, and these signals have been widely observed throughout the brain and are widely accepted as contributing to learning of stimulus-reward associations. However, it is not yet known how variables representing central moments of reward probability distributions arise. For example, how many trials are required for a neuron to assign an accurate statistical value to a stimulus based on the history of rewards associated with that stimulus? A recent study provides insight to this mechanism, showing that dopamine neurons acquire predictive value signal coding from the frequency of rewards [27]. More studies are required to further elucidate the generation of predictive coding signals in other brain areas including the orbitofrontal cortex.

### 4.2 How are these objective values combined with subjective values?

Decision makers’ choices typically do not represent objective, statistical information of uncertain rewards linearly. They tend to distort reward signals, as in risk-seeking or risk-averse behaviour, which represent non-linear transformations of risk (variance) information [28]. There is also a tendency to overestimate low probabilities and underestimate high probabilities, reflecting distortions of reward probabilities [[29], [30], [31]]. Distortions of both risk and reward probability result in non-linear transformations of value signals. These observations clearly show that reward signals are not transmitted through the nervous system linearly, as they are not expressed this way in behaviour of revealed preferences. Yet, objective reward parameters are represented in the brain through coding at the single neuron level. So what happens to these objective representations between the coding stage and behavioural expression? Recent studies have shown that subjective values of reward signals are indeed also represented through coding at the single neuron level. For example, the dopamine prediction error signal appears to be derived from a subjective rather than an objective reference point [32,33]. Likewise, reward value signals in orbitofrontal neurons also represents subjective values [7,25,34]. Therefore, both objective and subjective values are coded by single neurons in the orbitofrontal cortex and other brain regions. It is not yet clear how exactly these objective and subjective values are combined to drive observed behaviour.

### 4.3 Crosstalk between areas

Finally, many studies provide correlational evidence from single neurons and causal evidence from lesion studies identifying the involvement of several brain areas in coding objective and subjective values relevant for decision making. However, it is clear that there is communication between these brain areas, and we do not yet know what are the key components of communication between these areas that give rise to behaviour and in particular distortion of learned reward values as revealed through behavioural preferences. Future studies will benefit from recordings in multiple areas simultaneously to identify the relative contribution of different areas during learning and decision making under conditions of uncertainty.
